# Mapping sex-based multimorbidity networks in type 1 diabetes: a real-world study from Shanghai

**DOI:** 10.3389/fendo.2026.1712484

**Published:** 2026-02-18

**Authors:** Yuxuan Sheng, Ping He, Junlei Su, Zhongyun Zhang, Tingyu Zhang, Haolei Pan, Huayan Yao, Yanbin Xue, Xueyuan Li, Bin Cui, Zizheng Zhang, Weiqiong Gu

**Affiliations:** 1 Department of Endocrine and Metabolic Diseases, Shanghai Institute of Endocrine and Metabolic Diseases, Ruijin Hospital, Shanghai Jiao Tong University School of Medicine, Shanghai, China; 2 Shanghai National Clinical Research Center for Metabolic Diseases, Key Laboratory for Endocrine and Metabolic Diseases of the National Health Commission of the PR China, Shanghai Key Laboratory for Endocrine Tumor, State Key Laboratory of Medical Genomics, Ruijin Hospital, Shanghai Jiao Tong University School of Medicine, Shanghai, China; 3 Link Healthcare Engineering and Information Department, Shanghai Hospital Development Center, Shanghai, China; 4 Computer Net Center, Ruijin Hospital, Shanghai Jiao Tong University School of Medicine, Shanghai, China; 5 Wonders Information Co. Ltd., Shanghai, China

**Keywords:** multimorbidity, network analysis, real-world data, sex differences, type 1 diabetes mellitus, type 2 diabetes mellitus

## Abstract

**Background:**

Type 1 diabetes mellitus (T1DM) is a chronic autoimmune disorder associated with a substantial metabolic burden. However, large-scale, real-world studies assessing sex-specific multimorbidity patterns in T1DM remain limited.

**Objective:**

To characterize the clinical profiles and multimorbidity patterns in patients with T1DM, using age- and sex-matched individuals with type 2 diabetes mellitus (T2DM) as comparators.

**Methods:**

Using the Shanghai Hospital Link Database, we identified patients aged 10–79 years with a first T1DM-coded diagnosis between 2014 and 2023 and selected 1:1 sex- and index-age–matched T2DM comparators. Clinical characteristics were compared between groups overall and stratified by sex. Sex-stratified multimorbidity networks were constructed and analyzed using standard network metrics. Subgroup-unique comorbid disease pairs were identified using the Salton Cosine Index.

**Results:**

We included 18,971 patients with T1DM and 18,971 matched T2DM comparators. Compared with T2DM, T1DM showed shorter observed follow-up, higher glycated hemoglobin, higher high-density lipoprotein cholesterol, and lower triglyceride, with more pronounced differences among females. Hypertension and dyslipidemia were the most central comorbidities across all networks, closely linked to cardiovascular and cerebrovascular conditions. The female T1DM network exhibited greater complexity and connectivity than the male T1DM network. Subgroup-unique pairs were more frequent in T1DM, including peripheral vascular disease–nonalcoholic fatty liver disease and diabetic eye complications–stroke in males, and neuropathy–osteoporosis, nephropathy–chronic gastritis, and chronic gastritis–asthma in females.

**Conclusion:**

We identified shared cardiometabolic comorbidities (hypertension and dyslipidemia) across diabetes types and sexes, and sex-specific multimorbidity co-occurrence signatures in T1DM. These findings support integrating blood pressure and lipid optimization with glycemic management, and underscore the need for sex-sensitive surveillance of subgroup-specific clusters, highlighting broader multisystem clustering in females and vascular-risk co-occurrence signals in males that may warrant intensified multifactorial atherosclerotic cardiovascular disease prevention.

## 1 Introduction

Diabetes mellitus is a major chronic metabolic disorder and poses a growing global public health challenge. Type 1 diabetes mellitus (T1DM), a lifelong autoimmune disease affecting an estimated 9.5 million people worldwide (1), is characterized by abrupt onset and significant glycemic variability, which predisposes patients to an elevated risk of both acute and chronic complications. Acute complications primarily include diabetic ketoacidosis and severe hypoglycemia, whereas chronic complications involve microvascular disorders (such as diabetic retinopathy, nephropathy, and neuropathy) as well as macrovascular diseases (including cardiovascular and peripheral vascular disease) (2–6).

It is important to recognize that diabetes seldom occurs in isolation. Patients often present with multiple chronic conditions, forming distinct multimorbidity patterns (7). In assessing these patterns, real-world studies (RWSs) offer unique advantages over randomized controlled trials (RCTs). Although RCTs remain the gold standard for evaluating therapeutic efficacy due to their high internal validity and robust bias control, they typically focus on disease-specific outcomes and frequently exclude patients with complex multimorbidity. As a result, RCTs provide limited insight into the broader landscape of comorbidities and may lack generalizability to real-world clinical populations (8–10). In contrast, RWSs include more heterogeneous and clinically representative patient groups, enabling a more accurate characterization of comorbidity profiles and the true burden of multimorbidity in routine diabetes care (9, 11, 12).

Network analysis provides an innovative and structured framework for investigating multimorbidity in diabetes. This approach facilitates the identification of hub diseases, inter-disease associations, and potential disease progression pathways. For example, Aguado et al. (13) constructed a comorbidity network for type 2 diabetes mellitus (T2DM) using electronic health records (EHR) from over 3 million adults in Catalonia, revealing complex patterns of co-occurrence among chronic conditions. Zhang et al. (11) developed sex- and age-stratified comorbidity networks based on electronic medical records from nearly 500,000 Chinese patients with T2DM, highlighting distinct comorbidity structures across demographic subgroups. Together, these studies demonstrate the value of network-based methods in elucidating the architecture of multimorbidity and advancing our understanding of disease interrelationships in diabetes.

However, most existing real-world studies have focused predominantly on T2DM, limiting the generalizability of their findings to the T1DM population. The limited research on comorbidities in T1DM has primarily examined single-organ complications, such as cardiovascular or renal diseases, without offering a comprehensive overview (7). To date, no study has explicitly applied network analysis to systematically characterize multimorbidity patterns in T1DM. Furthermore, few investigations have integrated real-world data, network analysis, and sex-stratified approaches to explore clinical encounter patterns in T1DM.

## 2 Materials and methods

### 2.1 Data sources

All data in this study were sourced from the Shanghai Hospital Link Database (SHLD), a large-scale regional clinical database that aggregates general clinical information from 37 tertiary hospitals across Shanghai and covers more than 99% of the city’s residents. This system enables comprehensive tracking of healthcare encounters, including patient demographics, timestamps of visits, and diagnostic codes based on the International Classification of Diseases, 10th Revision (ICD-10). The SHLD has been available for academic research on diabetes since 2018, with data access granted upon appropriate review and approval. The study protocol was approved by the Ethics Committee of Ruijin Hospital (Approval No. 2024-03), which granted a waiver of informed consent due to the use of de-identified data.

### 2.2 Study population and design

We included patients aged 10–79 years with a first recorded T1DM-coded diagnosis record in the SHLD between January 1, 2014 and December 31, 2023. The index date was defined as the earliest date on which a patient had any T1DM-coded diagnosis record in SHLD, identified by ICD-10 codes or corresponding clinical diagnoses, including codes for T1DM with specified complications. Analogous rules were applied to identify T2DM records and define the index date. A comparison cohort of patients with T2DM was selected using 1:1 exact matching on sex and index age (in years). “Date of last visit” was defined as the most recent clinical encounter recorded in SHLD, and “observed follow-up” was calculated as the time interval between the index date and the last recorded visit. The final study cohort comprised 18,971 patients with T1DM and 18,971 matched patients with T2DM. Laboratory parameters were extracted from each patient’s most recent available recorded visit during the observed follow-up period.

### 2.3 Statistical analysis

Descriptive statistics were used to summarize demographic characteristics, observed follow-up, and laboratory parameters. Continuous variables were summarized as mean ± standard deviation (SD) for approximately normally distributed data and as median (interquartile range, IQR) for skewed data; categorical variables were summarized as counts (percentages). Between-group differences were assessed using standardized mean differences (SMDs), with an absolute SMD < 0.10 indicating a small difference. For skewed continuous variables, SMDs were calculated on log-transformed values (natural log); when zero values were present, a log(x+1) transformation was applied. Analyses were conducted for the overall T1DM and T2DM cohorts and stratified by sex (male and female subgroups). All analyses were performed using R (version 4.5.0).

### 2.4 Multimorbidity

Multimorbidity was defined as the presence of two or more conditions (excluding diabetes itself) recorded after the index date. Diagnoses were restricted to ICD-10 Chapters I–XIV, which primarily capture well-defined diseases and exclude chapters dominated by symptoms/signs and external causes. To ensure clinical relevance and stable estimation, conditions were retained if their prevalence was ≥1% within the T1DM and T2DM cohort, separately. Conditions meeting these criteria were retained for analysis (**Supplementary Table S1**).

### 2.5 Multimorbidity network

The multimorbidity network consisted of nodes and edges. Nodes represented comorbid conditions identified in patients with T1DM or T2DM, and edges represented the co-occurrence between pairs of diseases. Edge strength was quantified using the Salton Cosine Index (SCI), with higher values indicating a greater likelihood of co-occurrence and stronger clinical relevance.

Separate networks were constructed for male and female subgroups within both the T1DM and T2DM cohorts. Network topology was characterized using standard metrics, including degree, weighted degree, density, and centrality measures. Degree refers to the number of direct connections per node, while weighted degree represents the sum of SCI values for those connections, reflecting cumulative association strength. Network density was defined as the ratio of observed edges to the maximum possible number of edges, indicating overall connectivity. Closeness centrality, defined as the inverse of the average shortest path from a given node to all others, was replaced by harmonic centrality, which provides a comparable measure of node proximity and is applicable to networks that are not fully connected.

Hub diseases were defined as the top 10 comorbid conditions ranked by PageRank value (14). These diseases were more common in patients with complex comorbidity profiles or presumed to exert a disproportionate influence on the overall multimorbidity burden.

### 2.6 SCI calculation and application

This study used SCI to quantify the strength of associations between pairs of comorbid conditions within the multimorbidity networks of patients with T1DM and T2DM. The SCI is advantageous in that it is unaffected by sample size, which is crucial for large-scale clinical data (15). For each subgroup, SCI is calculated using the following formula:

(1) $$SCI_{ij}=\frac{c_{ij}}{\sqrt{c_{i}c_{j}}}$$

(2) $$\Phi_{ij}=\frac{c_{ij}N\;-\;c_{i}c_{j}}{\sqrt{c_{i}c_{j}(N\;-\;c_{i})(N\;-\;c_{j})}}$$

(3) $$t_{ij}=\frac{\Phi_{ij}\sqrt{c_{ij}\;-\;2}}{\sqrt{1\;-{\;\Phi }_{ij}^{2}}}$$

Where $c_{ij}$ is the number of patients with both diseases i and j; $c_{i}$ and $c_{j}$ are the number of patients with diseases i and j, respectively; *N* is the total number of patients in the subgroup.

In addition to the SCI, we used the Phi coefficient (Φ) to further assess disease pair associations by incorporating the total number of patients and comorbid conditions (Equation 2). This coefficient adjusts for imbalances in disease prevalence, refining the measure of co-occurrence between disease pairs. We also calculated the t-value ( ${\text{t}}_{ij}$), which is defined as Equation 3. This t-value is useful in quantifying the statistical significance of the relationship between diseases, ensuring that the associations are not spurious.

To account for both positive and negative correlations that could impact SCI estimates, we determined a cut-off threshold for the SCI values using the relationship between the SCI and the phi coefficient. The basic principle of this approach is that the number of positively correlated diseases is equal in the networks constructed using both measures (16). The steps to calculate this threshold are as follows: (i) For each pair of diseases in the study, calculate the SCI (Equation 1) and the Φ (Equation 2); (ii) Identify pairs with $c_{ij}$>0 and count the number of disease pairs ( q); (iii) Calculate the number of disease pairs ( e) that satisfy both p<0.05 ( $t_{ij}$>1.96; calculated in Equation 3) and $c_{ij}$> $\sum c_{ij}/q$; (iv) The final SCI cut-off is derived using the number of pairs e that satisfy the criteria, ensuring the multimorbidity network reflects the strongest, most statistically significant associations.

## 3 Results

### 3.1 Comparative clinical characteristics of patients with T1DM and T2DM

**Table 1** summarizes the descriptive statistics for the overall study population and sex-stratified subgroups. Compared with patients with T2DM, those with T1DM had a shorter observed follow-up (median [IQR], 3.80 [1.36, 7.13] vs. 8.28 [4.85, 10.34] years; SMD = −0.59) and higher mean glycated hemoglobin (HbA1c) (63 ± 23 vs. 58 ± 20 mmol/mol; SMD = 0.23). In lipid measures, patients with T1DM exhibited higher high-density lipoprotein cholesterol (HDL-C) (1.35 ± 0.44 vs. 1.22 ± 0.37 mmol/L; SMD = 0.33) and lower triglycerides (TG) (median [IQR], 1.12 [0.78, 1.72] vs. 1.41 [0.99, 2.09] mmol/L; SMD = –0.36) than those with T2DM.

**Table 1:** Clinical characteristics of patients with T1DM and T2DM in the overall population and sex-stratified subgroups.

Characteristics	Overall			Male			Female		
	T1DM (N = 18,971)	T2DM (N = 18,971)	SMD	T1DM (n = 9,602)	T2DM (n = 9,602)	SMD	T1DM (n = 9,369)	T2DM (n = 9,369)	SMD
Age bands, n (%)									
10–17 years	686 (3.60)	686 (3.6)	<0.01	320 (3.3)	320 (3.3)	<0.01	366 (3.9)	366 (3.9)	<0.01
18–39 years	5,246 (27.7)	5,246 (27.7)	<0.01	2,499 (26.0)	2,499 (26.0)	<0.01	2,747 (29.3)	2,747 (29.3)	<0.01
40–59 years	6,232 (32.9)	6,232 (32.9)	<0.01	3,438 (35.8)	3,438 (35.8)	<0.01	2,794 (29.8)	2,794 (29.8)	<0.01
60–79 years	6,807 (35.9)	6,807 (35.9)	<0.01	3,345 (34.8)	3,345 (34.8)	<0.01	3,462 (37.0)	3,462 (37.0)	<0.01
Sex, Female, n (%)	9,369 (49.4)	9,369 (49.4)	<0.01	–	–	–	–	–	–
Index age (years)	49.67 ± 17.08	49.67 ± 17.08	<0.01	49.94 ± 16.50	49.94 ± 16.50	<0.01	49.39 ± 17.66	49.39 ± 17.66	<0.01
Observed follow-up (years)	3.80 (1.36, 7.13)	8.28 (4.85, 10.34)	−0.59	3.62 (1.22, 6.94)	8.18 (4.63, 10.33)	−0.59	3.96 (1.50, 7.32)	8.40 (5.09, 10.35)	−0.60
Laboratory parameters									
HbA1c, mmol/mol (NGSP, %)	63 ± 23 (7.9 ± 2.1)	58 ± 20 (7.4 ± 1.8)	0.23	64 ± 24 (8.1 ± 2.2)	60 ± 20 (7.6 ± 1.8)	0.22	60 ± 22 (7.7 ± 2.0)	55 ± 20 (7.2 ± 1.8)	0.25
TC (mmol/L)	4.69 ± 1.32	4.60 ± 1.26	0.07	4.48 ± 1.33	4.40 ± 1.25	0.06	4.91 ± 1.28	4.82 ± 1.23	0.07
LDL-C (mmol/L)	2.77 ± 1.02	2.74 ± 0.99	0.03	2.68 ± 1.02	2.63 ± 0.97	0.05	2.87 ± 1.02	2.86 ± 1.00	0.01
HDL-C (mmol/L)	1.35 ± 0.44	1.22 ± 0.37	0.33	1.23 ± 0.38	1.11 ± 0.32	0.33	1.48 ± 0.45	1.33 ± 0.39	0.36
TG (mmol/L)	1.12 (0.78, 1.72)	1.41 (0.99, 2.09)	−0.36	1.13 (0.79, 1.72)	1.39 (0.98, 2.07)	−0.33	1.12 (0.77, 1.72)	1.43 (1.00, 2.10)	−0.40
ALT (U/L)	18.20 (13.00, 27.00)	20.80 (14.00, 31.00)	−0.18	20.00 (14.00, 29.10)	22.65 (16.00, 34.00)	−0.20	17.00 (12.00, 24.00)	18.90 (13.00, 28.00)	−0.18
AST (U/L)	20.00 (15.00, 26.00)	20.00 (15.00, 27.00)	−0.10	20.00 (15.00, 26.56)	21.00 (15.30, 27.05)	−0.10	19.20 (14.00, 25.98)	20.00 (15.00, 26.40)	−0.10
ALP (U/L)	75.00 (60.30, 95.40)	73.00 (59.00, 91.57)	0.07	76.00 (62.00, 96.55)	73.00 (60.00, 90.00)	0.10	74.00 (59.00, 94.00)	73.00 (58.40, 93.00)	0.03
GGT (U/L)	19.00 (13.00, 30.00)	22.00 (15.00, 36.00)	−0.22	21.60 (15.00, 34.00)	25.00 (17.50, 41.00)	−0.21	16.00 (12.00, 26.00)	20.00 (14.00, 31.30)	−0.24
UA (µmol/L)	301.05 (240.00, 374.02)	322.00 (261.00, 392.70)	−0.15	327.90 (265.80, 400.00)	349.00 (287.00, 416.10)	−0.13	274.30 (220.00, 341.20)	297.00 (241.10, 362.00)	−0.18
UMA (mg/L)	11.28 (7.20, 40.50)	16.80 (10.00, 58.00)	−0.14	12.80 (7.91, 56.20)	19.00 (10.20, 73.68)	−0.11	11.00 (6.60, 29.30)	14.84 (10.00, 44.30)	−0.18

Continuous variables are presented as mean ± SD for approximately normally distributed data and as median (IQR) for skewed data; categorical variables are presented as counts (percentages). Percentages may not total 100% because of rounding. Between-group differences are assessed using SMDs, with an absolute SMD < 0.10 indicating a small difference. For skewed continuous variables, SMDs are computed on log-transformed values (log[x+1] when zeros are present). HbA1c is reported primarily as IFCC (mmol/mol, no decimals), followed by NGSP (%) with one decimal place.

SMD, standardized mean difference; SD, standard deviation; IQR, interquartile range; IFCC, International Federation of Clinical Chemistry and Laboratory Medicine; NGSP, National Glycohemoglobin Standardization Program; HbA1c, glycated hemoglobin; TC, total cholesterol; LDL-C, low-density lipoprotein cholesterol; HDL-C, high-density lipoprotein cholesterol; TG, triglyceride; ALT, alanine aminotransferase; AST, aspartate aminotransferase; ALP, alkaline phosphatase; GGT, gamma-glutamyl transferase; UA, uric acid; UMA, urinary microalbumin.

Sex-stratified analyses revealed that these between-group differences were more pronounced in females. Specifically, females with T1DM had a shorter observed follow-up (median [IQR], 3.96 [1.50, 7.32] vs. 8.40 [5.09, 10.35] years; SMD = –0.60), higher HbA1c (60 ± 22 vs. 55 ± 20 mmol/mol; SMD = 0.25), higher HDL-C (1.48 ± 0.45 vs. 1.33 ± 0.39 mmol/L; SMD = 0.36), and lower TG (median [IQR], 1.12 [0.77, 1.72] vs. 1.43 [1.00, 2.10] mmol/L; SMD = –0.40), compared to females with T2DM. These differences were more substantial than those observed in males.

### 3.2 Comorbidity characteristics in patients with T1DM and T2DM

#### 3.2.1 Multimorbidity network characteristics in sex-stratified subgroups

**Table 2** summarizes network metrics for the sex-stratified multimorbidity networks. Among the four networks, the female T1DM network had the highest number of edges, while the female T2DM network had the highest density, average degree, average weighted degree, and average harmonic centrality, and had the fewest node. Within T1DM, the female network had higher density, average degree, average weighted degree, and average harmonic centrality than the male network. In sex-specific comparisons between diabetes types, the male T1DM network had higher density, average degree, average weighted degree, and average harmonic centrality than the male T2DM network. In contrast, the female T1DM network showed lower values for these metrics than the female T2DM network.

**Table 2:** Network metrics of sex-stratified multimorbidity networks in patients with T1DM and T2DM.

Metrics	T1DM		T2DM	
	Male	Female	Male	Female
Nodes	22	22	22	20
Edges	82	92	78	89
Density	0.355	0.398	0.338	0.468
Average Degree	7.455	8.364	7.091	8.900
Average Weighted Degree	2.780	3.041	2.623	3.200
Average Harmonic Centrality	0.177	0.213	0.173	0.254

Sex-stratified multimorbidity networks for T1DM and T2DM were visualized (**Supplementary Figure S1**). Across all networks, hypertension and dyslipidemia were the most central comorbidities, exhibiting the highest degree and/or PageRank values (**Supplementary Tables S2, S3**), and were strongly connected with other cardiovascular and cerebrovascular diseases, such as ischemic heart disease and cerebrovascular disease (**Supplementary Tables S4, S5**). At the system level, circulatory diseases showed the highest total degree and weighted degree, accounting for the greatest system-level connectivity (**Supplementary Table S6**). Subgroup-specific patterns were also observed; for example, the co-occurrence of chronic hepatitis and cirrhosis was detected only in the male T1DM, female T1DM, and male T2DM networks. In contrast, anxiety or depression appeared exclusively in the female T1DM and female T2DM networks, suggesting potential sex-related differences in comorbidity profiles or clinical recognition.

#### 3.2.2 Unique and significant co-occurring disease pairs in sex-stratified multimorbidity networks

**Table 3** summarizes significant co-occurring disease pairs that were uniquely identified in each sex-specific network (i.e., present in one diabetes–sex subgroup but absent in the other three subgroups). In the male T1DM network, two unique significant pairs were identified: diabetic eye complications with stroke and peripheral vascular disease (PVD) with nonalcoholic fatty liver disease (NAFLD). In the female T1DM network, three unique pairs were observed: diabetic nephropathy with chronic gastritis, diabetic neuropathy with osteoporosis, and chronic gastritis with asthma. No unique significant pairs were detected in the male T2DM network, whereas the female T2DM network exhibited one unique pair, hypertension with asthma. Overall, unique significant pairs were more frequently observed in T1DM, particularly among females, suggesting sex- and diabetes-type–specific clustering patterns in multimorbidity.

**Table 3:** Unique and significant co-occurring disease pairs in the sex-stratified multimorbidity networks of T1DM and T2DM.

Type	Co-occurring disease pairs	SCI
T1DM (male)	Diabetic Eye Complications -– Stroke	0.245
	PVD -– NAFLD	0.246
T1DM (female)	Diabetic Nephropathy -– Chronic Gastritis	0.249
	Diabetic Neuropathy -– Osteoporosis	0.293
	Chronic Gastritis -–Asthma	0.253
T2DM (male)	None	None
T2DM (female)	Hypertension -– Asthma	0.250

SCI, Salton cosine index; PVD, peripheral vascular disease; NAFLD, nonalcoholic fatty liver disease.

**Figure 1:**
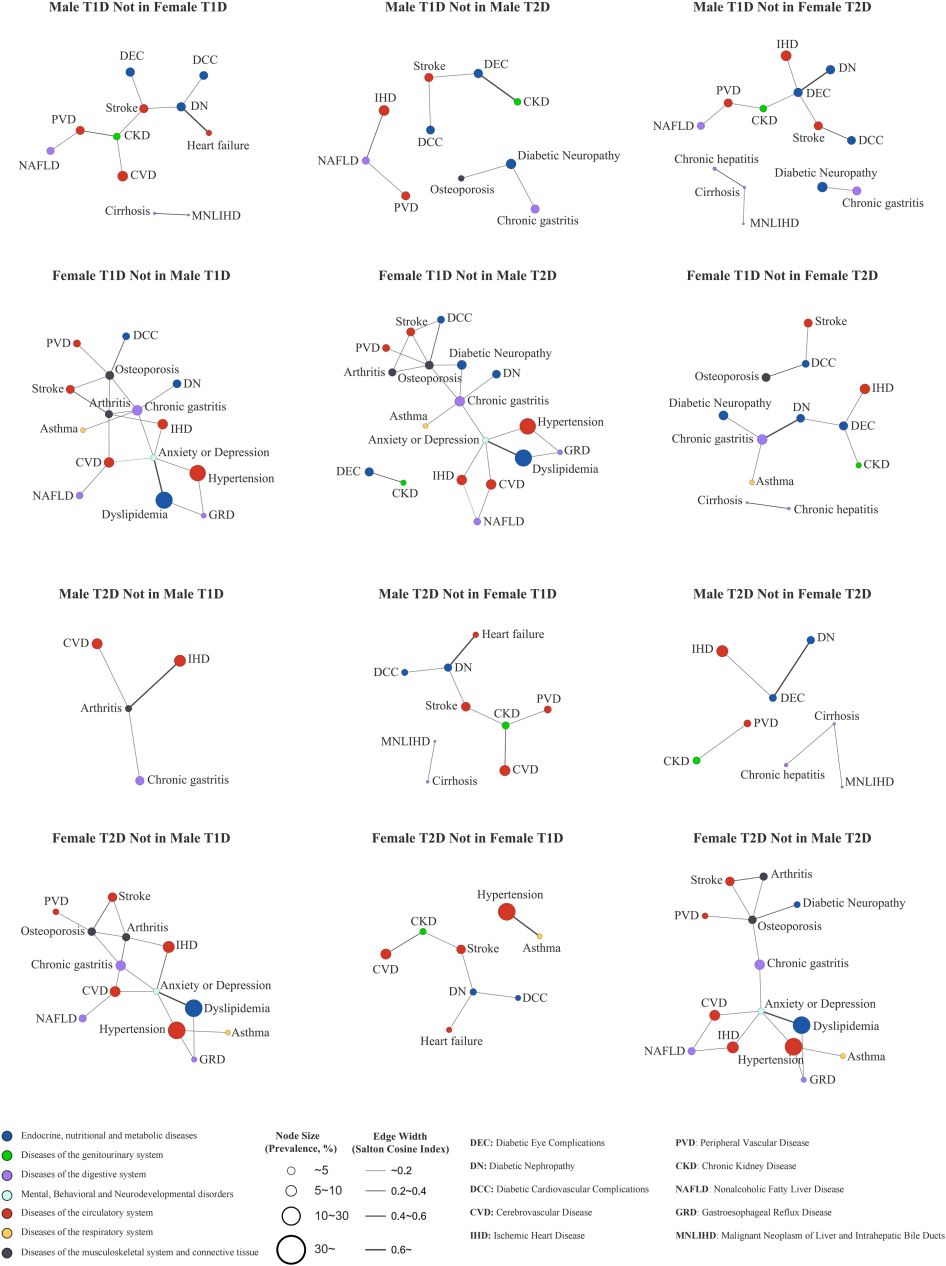
Contrast-based difference networks (A-not-in-B). Contrast-based difference networks (A-not-in-B) showing co-occurring disease pairs present in the index subgroup network but absent in the comparison network.

**Figure 1** visualizes contrast-based difference networks (A-not-in-B), highlighting co-occurring disease pairs that are present in one subgroup network but absent in a comparison network. We observed that female-specific contrasts tended to yield larger difference sub-networks (i.e., more subgroup-specific edges) than male-specific contrasts. Comparisons spanning both sex and diabetes type also produced more subgroup-specific edges than contrasts that differed by sex alone or diabetes type alone. Notably, although hypertension and dyslipidemia were highly central in all four primary networks, edges involving these two nodes appeared predominantly in female-specific difference networks, suggesting sex-related differences in connectivity profiles within the cardiometabolic multimorbidity cluster.

## 4 Discussion

Utilizing clinical data from the SHLD, we mapped sex-stratified multimorbidity networks in T1DM and contrasted them with age- and sex-matched T2DM comparators, providing a systems-level view of multimorbidity co-occurrence in real-world care. Overall, two findings stood out: hypertension and dyslipidemia were the most central comorbidities across all sex–diabetes subgroups, and contrast-based analyses revealed pronounced subgroup-specific co-occurrence patterns, including male- and female-specific co-occurring pairs. Higher HbA1c levels observed in T1DM than in matched T2DM provide clinical context for a higher glycemic burden and a complication-prone background in which these co-occurrence patterns arise.

Network analysis of large-scale EHR data can quantify disease co-occurrence, identify highly connected conditions and co-occurring disease pairs (17, 18), and enables standardized comparisons of multimorbidity structure across populations within a unified analytical pipeline (19, 20). Accordingly, we constructed four subgroup networks to characterize sex- and diabetes type–specific differences in comorbidity connectivity.

In all four subgroup networks, hypertension and dyslipidemia were the most central comorbidities across both diabetes types and sexes, co-occurring broadly with other comorbidities, particularly cardiovascular diseases. Clinically, this consistent central status highlights the importance of sustained attention to blood pressure control and lipid management in T1DM, alongside glycemic management, in a manner aligned with cardiovascular prevention strategies routinely emphasized in T2DM. Evidence from large-scale meta-analyses supports meaningful reductions in major cardiovascular events with antihypertensive therapy and LDL-C lowering (21, 22). In light of the substantial burden of cardiovascular morbidity in T1DM (23, 24), anchoring integrated care around these cardiometabolic hubs may be especially important for mitigating long-term cardiovascular risk. From a health-systems perspective, these findings support prioritizing blood pressure and lipid control as measurable quality measures in T1DM care; they may also inform integrated care pathways centered on high-centrality cardiometabolic conditions, thereby facilitating scalable implementation of atherosclerotic cardiovascular disease (ASCVD) prevention.

Sex-stratified network analysis further enabled the identification of subgroup-unique co-occurring pairs that may be overlooked in routine practice. In the T1DM networks, we identified two male-specific pairs (NAFLD–PVD, diabetic eye complications–stroke) and three female-specific pairs (neuropathy–osteoporosis, nephropathy–chronic gastritis, and chronic gastritis–asthma), highlighting sex heterogeneity in multimorbidity co-occurrence.

In our EHR coding, PVD represents a broad peripheral vascular disease label. Since much of the existing literature reports peripheral arterial disease (PAD)-specific outcomes, an atherosclerotic subtype within the broader PVD spectrum, we cite PAD-focused studies as the closest clinical counterpart while acknowledging phenotypic heterogeneity. In diabetes, including T1DM, NAFLD has been associated with a higher prevalence of PAD/PVD (25, 26). Proposed links include shared cardiometabolic disturbances such as insulin resistance, atherogenic dyslipidemia, and systemic inflammation, which may promote atherogenesis (26, 27). Accordingly, in males with T1DM, the identification of either NAFLD or PVD may serve as a clinical red flag that could prompt intensified ASCVD risk assessment and optimization of cardiometabolic factors, including lipids, within a comprehensive prevention framework.

We also observed a male-specific co-occurrence between diabetic eye complications and stroke. Robust longitudinal evidence in T1DM supports an association between diabetic retinopathy (DR) and incident stroke, and further indicates a severity-gradient pattern in which stroke incidence increases with DR severity (28–30). Mechanistically, DR may reflect cumulative systemic microvascular injury and endothelial dysfunction relevant to cerebrovascular vulnerability (31). Clinically, the identification of diabetic eye complications (including DR) in T1DM, especially in males, may therefore warrant heightened attention to multifactorial vascular risk assessment and management. Taken together, these male-specific pairs suggest that cardiometabolic/atherosclerotic phenotypes (e.g., NAFLD and PAD/PVD) and microvascular phenotypes (e.g., DR) may jointly characterize a vascular-risk co-occurrence signature in males with T1DM, emphasizing the need for integrated ASCVD prevention that includes lipid optimization alongside glycemic control.

Female-specific patterns suggested a more heterogeneous, multisystem multimorbidity profile in T1DM. In the primary networks, the female T1DM network exhibited a greater edge count and consistently higher connectivity across key network metrics than the male T1DM network, indicating a more interconnected co-occurring structure. Importantly, the contrast-based difference networks further showed that female-specific contrasts tended to yield larger sets of subgroup-specific edges than male-specific contrasts, and that edges involving hypertension and dyslipidemia appeared predominantly in female-specific difference networks, pointing to sex-differential connectivity profiles within the cardiometabolic cluster. Beyond this, female-specific pairs formed cross-system modules in the contrast-based difference networks, clustering around gastrointestinal and skeletal nodes and connecting to microvascular and respiratory/mental health conditions. Together, these findings suggest that sex differences may extend beyond overall multimorbidity burden to the composition of cardiometabolic connectivity and the presence of broader multisystem clusters.

This pattern is consistent with real-world evidence that females with T1DM experience a greater excess risk of cardiovascular events than males even with a more favorable cardiometabolic risk factor profile (32, 33). Moreover, a recent review highlighted that the usual female cardioprotection is attenuated in T1DM, with hypertension and diabetic kidney disease exerting a particularly strong influence on cardiovascular risk in females (34). Potential contributors include differences in disease spectra, screening/diagnostic ascertainment, treatment patterns, and healthcare utilization. Notably, therapeutic inertia may be more pronounced in females with T1DM (35), which could plausibly contribute to sex-differential cardiometabolic connectivity captured in EHR-derived networks. In addition, females with T1DM carry a greater multimorbidity burden, particularly autoimmune multimorbidity (7, 36), aligning with the broader multisystem co-occurrence patterns observed here. Collectively, our findings support heightened awareness of potentially more integrated, multisystem care needs in females with T1DM and motivate further longitudinal work to clarify the drivers and clinical implications of sex-differential connectivity.

A key strength of this study is the application of sex-stratified network analysis to a large-scale T1DM cohort with age- and sex-matched T2DM comparators. This systems-level approach complements conventional single-endpoint complication studies and leverages multi-hospital EHR data for comprehensive diagnosis capture in specialist care settings. However, several limitations should be considered. First, diagnoses were derived from ICD-10 codes, and some misclassification is inevitable, particularly in adult-onset diabetes where latent autoimmune diabetes in adults (LADA) or hybrid phenotypes may blur boundaries between T1DM and T2DM. Second, network edges represent co-occurrence only and do not account for temporality, directionality, or causality. Third, EHR-based capture may be incomplete and subject to differential ascertainment (e.g., care outside the SHLD tertiary-hospital system, missing laboratory measurements, and variation in screening/utilization), while secular changes in coding and guideline-driven screening over time may affect network structure. Finally, as our data were derived from tertiary-care outpatient and inpatient settings, generalizability to community care may be limited. Future work should validate key findings using time-stratified and longitudinal designs and assess robustness across alternative association measures.

## 5 Conclusions

This study provides a sex-stratified, real-world map of multimorbidity co-occurrence in T1DM, complementing conventional single-endpoint complication studies with a systems-level perspective. Across diabetes types and sexes, hypertension and dyslipidemia were the most central comorbidities in the primary networks, supporting blood pressure and lipid optimization, alongside glycemic control, as core components of T1DM management. Contrast-based difference networks further highlighted pronounced subgroup heterogeneity. In females, these findings suggest a need for more integrated surveillance that spans central cardiometabolic conditions and broader cross-system clusters. In males, vascular-risk co-occurrence signatures may warrant intensified, multifactorial ASCVD risk assessment and prevention, with lipid optimization as a particularly actionable component. These findings may help prioritize clinically coherent condition clusters for coordinated management. Longitudinal and implementation studies are needed to evaluate whether incorporating these co-occurrence signatures improves risk stratification and clinical outcomes in T1DM.

## Data Availability

The original contributions presented in the study are included in the article/**Supplementary Material**. Further inquiries can be directed to the corresponding authors.

## Supplementary material

Supplementary Figure 1 Multimorbidity networks stratified by sex in patients with T1DM and T2DM. **(a)** Male T1DM network. **(b)** Female T1DM network. **(c)** Male T2DM network. **(d)** Female T2DM network.
