# Supplementary figures and images for: Mapping sex-based multimorbidity networks in type 1 diabetes: a real-world study from Shanghai
Source: Front Endocrinol (Lausanne). 2026 Feb 18;17:1712484. doi: 10.3389/fendo.2026.1712484 (PMC12957172; doi:10.3389/fendo.2026.1712484)

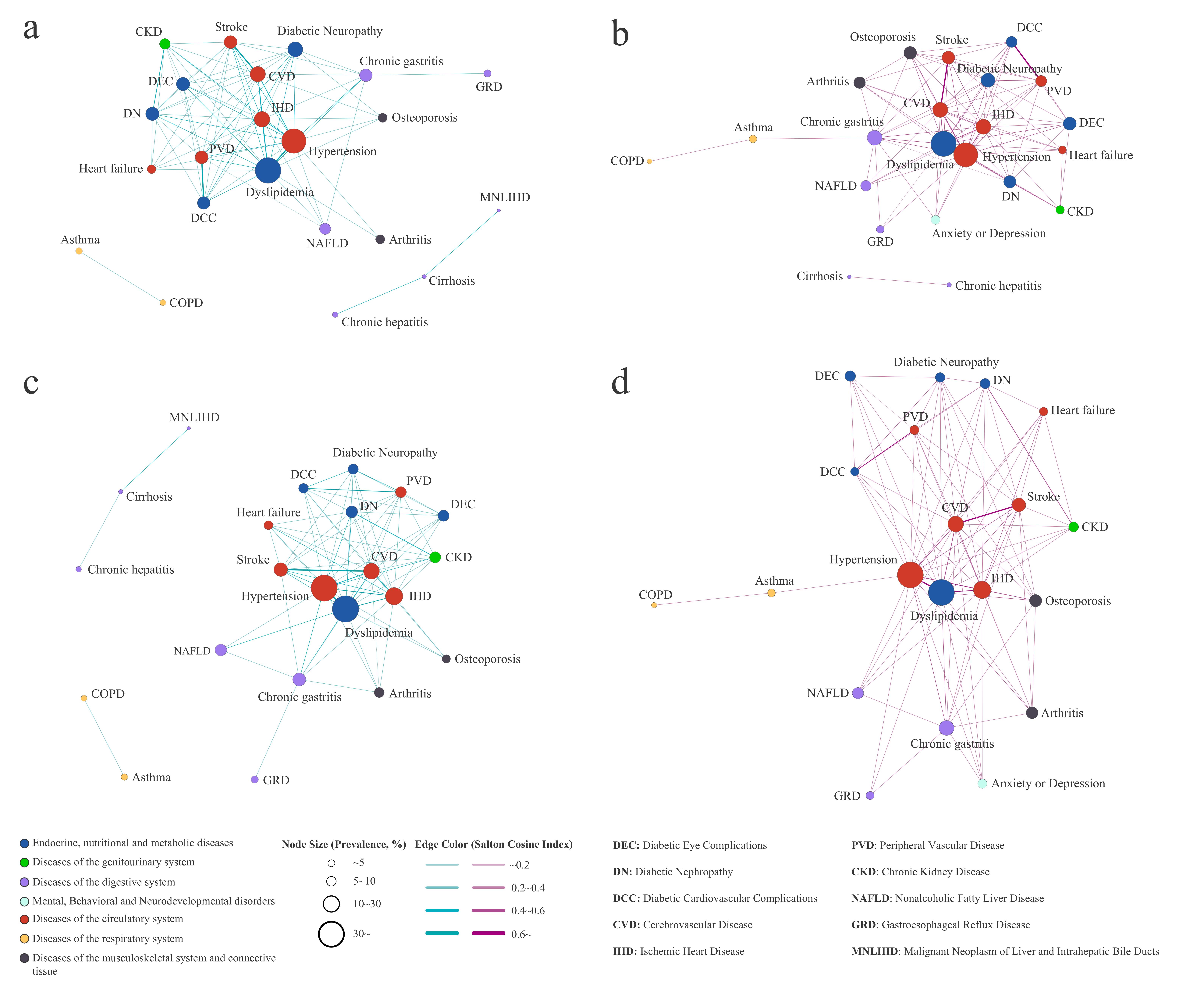

Supplement: Supplementary Figure 1 — Multimorbidity networks stratified by sex in patients with T1DM and T2DM. (a) Male T1DM network. (b) Female T1DM network. (c) Male T2DM network. (d) Female T2DM network. [file Image1.jpg]
